# 2,4-D and IAA Amino Acid Conjugates Show Distinct Metabolism in *Arabidopsis*

**DOI:** 10.1371/journal.pone.0159269

**Published:** 2016-07-19

**Authors:** Luděk Eyer, Thomas Vain, Barbora Pařízková, Jana Oklestkova, Elke Barbez, Hana Kozubíková, Tomáš Pospíšil, Roksana Wierzbicka, Jürgen Kleine-Vehn, Milan Fránek, Miroslav Strnad, Stéphanie Robert, Ondrej Novak

**Affiliations:** 1 Department of Virology, Veterinary Research Institute, Brno, Czech Republic; 2 Department of Forest Genetics and Plant Physiology, Umeå Plant Science Centre, Swedish University of Agricultural Sciences, Umeå, Sweden; 3 Laboratory of Growth Regulators, Centre of the Region Haná for Biotechnological and Agricultural Research, Institute of Experimental Botany CAS & Faculty of Science of Palacký University, Olomouc, Czech Republic; 4 Department of Applied Genetics and Cell Biology, University of Natural Resources and Life Sciences (BOKU), Vienna, Austria; 5 Department of Chemical Biology and Genetics, Centre of the Region Haná for Biotechnological and Agricultural Research, Faculty of Science of Palacký University, Olomouc, Czech Republic; Iwate University, JAPAN

## Abstract

The herbicide 2,4-D exhibits an auxinic activity and therefore can be used as a synthetic and traceable analog to study auxin-related responses. Here we identified that not only exogenous 2,4-D but also its amide-linked metabolite 2,4-D-Glu displayed an inhibitory effect on plant growth via the TIR1/AFB auxin-mediated signaling pathway. To further investigate 2,4-D metabolite conversion, identity and activity, we have developed a novel purification procedure based on the combination of ion exchange and immuno-specific sorbents combined with a sensitive liquid chromatography-mass spectrometry method. In 2,4-D treated samples, 2,4-D-Glu and 2,4-D-Asp were detected at 100-fold lower concentrations compared to 2,4-D levels, showing that 2,4-D can be metabolized in the plant. Moreover, 2,4-D-Asp and 2,4-D-Glu were identified as reversible forms of 2,4-D homeostasis that can be converted to free 2,4-D. This work paves the way to new studies of auxin action in plant development.

## Introduction

The distribution of the phytohormone auxin (indole-3-acetic acid, IAA) mediates most aspects of plant development by triggering molecular processes, which control organogenesis in response to environmental and development cues. Auxin regulation of gene expression occurs by the action of the nuclear-localized F-box proteins TRANSPORT INHIBITOR RESPONSE 1/AUXIN SIGNALING F-BOX (TIR1/AFB), which promote the degradation of the AUXIN/INDOLE-3-ACETIC ACID (Aux/IAA) transcriptional repressors in an auxin-dependent manner via the ubiquitin-proteasome system (UPS) [1]. Loss of any components of the auxin signaling pathway such as the activity of the upstream elements AUXIN-RESISTANT-1 (AXR1) and CULLIN1 (CUL1) induces resistance to exogenous auxin application [2,3].

The cellular auxin level is regulated by auxin biosynthesis in conjunction with directional auxin transport, degradation and conversion to conjugated forms [4]. The proportion of free active IAA is highly regulated and kept at an optimum level within tissues and inside the cell [5]. Beside free IAA, two alternative forms of IAA arise: the ester-linked and amide-linked IAA conjugates [6], within which IAA-Alanine (IAA-Ala), IAA-Glutamate (IAA-Glu), IAA-Leucine (IAA-Leu) and IAA-Aspartate (IAA-Asp) are the predominant forms [7]. To maintain auxin homeostasis, the abundance of supposedly inactive IAA conjugates differs inside *Arabidopsis thaliana* organs and between ecotypes and is subject to complex regulation to compensate for metabolism change imposed by *SUPERROOT1* (*SUR1*) mutation or *YUCCA1* (*YUC1*) overexpression [8]. At least seven members of the GRETCHEN HAGEN 3 (GH3) protein family have been shown to be involved in IAA conjugation to amino acids and their expression is regulated by auxin [9,10]. This mechanism is reversible and the hydrolysis of IAA conjugates to free IAA is facilitated by the IAA-LEUCINE RESISTANT 1 (ILR1)-like amidohydrolase family [11–13]. Expression patterns of GH3 and ILR1-like genes reveal they might have tissue-specific functions [14,15]. Even though a lot has been recently discovered about IAA conjugate tissue distribution [8,16], the minimal amount of evidence for their bioactivity [17] and low number of associated mutants with a phenotype [14,15,18] have been shown. Therefore their biosynthesis process and biological function remain as complex standing questions in auxin biology.

Synthetic auxins have been used extensively to study auxin-related activities, with the advantage of being more stable than endogenous auxins. Such substances include the canonical 2,4-dichlorophenoxyacetic acid (2,4-D) and other chlorine-substituted phenoxyacetic acid derivatives, which are still the most widely used herbicides for efficient control of broad-leaved weeds in cereal crops and lawns[19,20]. Commonly used, 2,4-D displays an auxinic activity including efficient stimulation of cell division and general plant growth at low concentration, while application of concentrated 2,4-D is toxic for dicot development [21]. The TIR1/AFB machinery has been shown to be the dominant signaling pathway involved in 2,4-D action, with TIR1 being the preeminent receptor within the auxin-related F-Box protein family [22].

The metabolism of 2,4-D in plants shares common features with metabolism of auxins and is based on three main mechanisms: the degradation or chemical modification of the acetic acid side chain, the hydroxylation of the aromatic ring and the conjugation of the 2,4-D molecule, mainly with amino acids and glucose [23]. Most of these molecules are believed to be catabolic products of 2,4-D detoxification metabolism that induce no auxin response and have been found in a broad range of species (wheat, potato, radish, lettuce and apple) [24,25]. In transgenic plants engineered for 2,4-D tolerance, the enzymatic degradation leads to 2,4-dichlorophenol, a less phytotoxic compound than 2,4-D [26](. 2,4-D-Glutamic acid (2,4-D-Glu) and 2,4-D-Aspartic acid (2,4-D-Asp) are two major metabolites, which represent almost 25% of all amide-linked conjugates isolated from 2,4-D treated plants [27]. Furthermore, *in vivo* metabolic conversions of 2,4-D-Glu to free 2,4-D, ring-hydroxylated metabolites and conjugates with other amino acids were observed in soybean cotyledon callus tissues [28]. Biological properties of 2,4-D conjugated with D-amino acids, including stimulation of coleoptile elongation and growth of soybean root callus, have been later reported by Davidonis *et al*. (1982) [29].

The detection and quantification of 2,4-D and its metabolites in plant tissues is still very challenging due to their low abundance. Different herbicide multiresidue screening methods using gas chromatography (GC) and liquid chromatography (LC) linked to mass spectrometry (MS) were reported for the analysis of residual phenoxy acids in soil, water and foods of animal origin [30–32]. Koesukwiwat *et al*. (2008) [33] applied LC–MS for the analysis of phenoxy acid herbicide residues in rice based on commonly used liquid extraction/partition and dispersive solid-phase extraction (QuEChERS method). The same extraction approach was later employed in an effective simultaneous determination of five plant growth regulators in fruits [34]. To our knowledge, no specific method for isolation of 2,4-D and its metabolites using immunoaffinity chromatography (IAC) has been previously published. However, IAC is the most powerful method for purifying specific classes of growth regulators from complex plant matrices [35].

Using anti-2,4-D monoclonal antibodies (E2/G2), we describe here a novel IAC procedure for efficient isolation of 2,4-D and its conjugated metabolites. We also report the synthesis of two 2,4-D-amino acid conjugates (as synthetic auxin analogs), 2,4-D-Glu and 2,4-D-Asp, and examined their potency to affect root growth in *Arabidopsis* seedlings via the TIR1/AFB auxin-mediated signaling pathway. Further investigation using a sensitive mass spectrometry-based method reveal their activity via the quantification of the catabolic/conversion products of 2,4-D, 2,4-D-Glu and 2,4-D-Asp. These highly specific and sensitive methodologies led us to identify the rate of 2,4-D conversion and will facilitate the development of further approaches to associate plant development and the activity of conjugative enzymes.

## Materials and Methods

### Preparation of 2,4-D-amino acid conjugates

In order to use 2,4-D conjugates for our research, 2,4-D-Glu and 2,4-D-Asp were synthesized by two-step procedure: (a) preparation of dimethyl-2,4-dichlorophenoxyacetyl-aminodicarboxylates using free 2,4-D acid as a starting reagent and (b) hydrolysis of the formed dimethyl-dicarboxylates (esters) to 2,4-D-Glu and 2,4-D-Asp with LiOH (S1 Table; S2 Table).

2-(2,4-dichlorophenoxy)acetic acid (1 mmol) was dissolved in dry dioxane (6.6 ml) and dry ethyl acetate (3.3 ml). Hydrochloride of glutamic/aspartic acid dimethyl ester (1 mmol) was added to the reaction mixture and the mixture was cooled in an ice bath. Then *N*,*N*′-dicyclohexylcarbodiimide (1 mmol) and *N*-methylmorfoline (1 mmol) were added alternately. The reaction mixture was stirred for 2 h at 0°C and then filtered and the solid material was washed with EtOAc (3x20 ml). The filtrate was washed with 4% aqueous H_3_PO_4_ (2x10 ml), 5% aqueous NaHCO_3_ (2x10 ml) and brine (10 ml) and the organic layer was dried with sodium sulfate and filtered. The solvent was removed *in vacuo* and the residue was purified by column flash chromatography on silica (CH_3_Cl:EtOAc, ratio 8:2) to afford a white solid.

Dimethyl ester (0.54 mmol) was then dissolved in tetrahydrofuran (THF; 20 ml). Lithium hydroxide monohydrate (11.5 mmol) was dissolved in water (10 ml) and then added to the reaction mixture at room temperature. After two hours, Et_2_O (20 ml) was added and the organic layer was washed with saturated sodium bicarbonate (3x10 ml). Combined aqueous layers were acidified to pH 2–3 with KHSO_4_ solution and extracted with dichloromethane (DCM; 3x10 ml). Combined organic layers were dried with Na_2_SO_4_, filtered and evaporated *in vacuo*. The remaining material was purified by column chromatography on silica, eluting with DCM:MeOH:acetic acid (5:1+1% acetic acid) to afford a white solid.

Nuclear magnetic resonance (NMR) and high resolution mass spectrometry (HRMS) of the products were used to verify the structure of 2,4-D amino conjugates (see S1 Table; S2 Table). The final product purities were 92.6% and 98.6% of 2,4-D-Glu and 2,4-D-Asp, respectively. Importantly, free 2,4-D was not detected as a possible impurity in both new synthetic auxin analogs by ultra-high performance liquid chromatography–tandem mass spectrometry (UHPLC-MS/MS) method as described below.

### Plant material and growth conditions

All *Arabidopsis* mutants and transgenic lines employed in this study are in the Columbia (Col-0) background and have been described previously: *axr1-30* [36] (Hotton *et al*., 2011), *cul1-6* and the auxin reporter line pDR5::GUS [37]. Surface-sterilized seeds were sown on solid medium (half-strength Murashige and Skoog (0.5 MS), sucrose 1%, agar 0.7%, pH 5.7) and stratified for 2 days at 4°C. Plants were grown on vertically oriented plates under a 16 h photoperiod at 21–23°C.

### Chemical treatment

Nine-day-old seedlings of *Arabidopsis* were grown on medium supplemented with 2,4-D, 2,4-D-Glu and 2,4-D-Asp at the indicated final concentrations (0.05 μM and 0.5 μM). For pDR5::GUS expression experiments, five-day-old seedlings were treated for 5 hours with the indicated chemicals using a concentration of 10 μM. Stock solutions were 10 mM in 100% DMSO. For quantification measurements, treated and DMSO-treated (control) 9-day-old plants were harvested, weighed and immediately plunged into liquid nitrogen. All samples were stored at -70°C. For short-term metabolization study, seven-day-old seedlings of *Arabidopsis* ecotype Col-0 were incubated for 5 min, 30 min and 3 hours in solid media supplemented with 1 μM of IAA and 2,4-D, and 10 μM of IAA-Asp and 2,4-D-Asp. Seedlings were collected and washed extensively with water to minimize traces of the external compound. All samples were then extracted and analyzed by UHPLC-MS/MS as explained below. 2,4-D and its amino acid conjugates were also analyzed for their short- and long-term stability. Solid media were treated with compounds at the final concentrations 0.05, 0.5, 1 and 10 μM and transferred to microcentrifuge tubes. After 5 min, 30 min, 3 hours and 7 days of incubation in the growth chamber (16/8 h of light/dark, 23°C), the media (200 μl) were melted in a microwave oven, diluted by a factor of 10 and purified by the two-step purification method (see below).

### Histochemical analysis, image processing and statistical analysis

Five-day-old seedlings of *Arabidopsis* expressing pDR5:GUS were fixed in 80% acetone at -20°C for 20 min and washed with 0.1 M phosphate buffer (Na_2_HPO_4_/NaH_2_PO_4_) at pH 7; 0.1% triton X100; 10 mM EDTA; 0.5 mM potassium ferrocyanide; 0.5 mM potassium ferricyanide) (GUS buffer). Samples were transferred to the GUS staining solution (2 mM X-Gluc (Duchefa) in GUS buffer) for 30 min in the dark at 37°C. The staining reaction was stopped using 70% ethanol. Plants were rehydrated progressively and mounted in 50% glycerol. Samples were observed using differential interference contrast microscopy with a Zeiss Axioplan microscope.

Primary root lengths were measured on seven-day-old seedlings using ImageJ software (W. Rasban, National Institutes of Health, Bethesda, MD, http://rsbweb.nih.gov/ij/) and statistical analyses of data (ANOVA and Tukey’s test) were performed using R software (John Chambers and colleagues, Bell laboratories).

### Extraction and purification of 2,4-D metabolites

For quantification of 2,4-D and its metabolites, 15–20 mg fresh weight of treated plant tissues were extracted in 1 ml of cold sodium phosphate buffer (50 mM, pH 7.0) according to the method previously described [8]. In each extract, 100 pmol of [^2^H_5_]-2,4-D (CDN Isotopes, Canada) and 10pmol of [^13^C_2_,^15^N]-2,4-D-Asp and [^13^C_2_,^15^N]-2,4-D-Glu synthesised from [^13^C_2_]-2,4-D and [^15^N]-Asp/[^15^N]-Glu as described above for non-labeled conjugates were added as internal standards to validate the determination. The samples were purified using a mixed mode reversed phase/strong anion exchange column (Oasis^®^ MAX, 1 ml/30 mg, Waters) followed by immunoaffinity chromatography (S1 Fig).

### Antibody characterization and immunoaffinity column preparation

The E2/G2 antibodies were prepared previously by Fránek *et al*. (1994) [38]. The binding properties and cross reactivity of the monoclonal E2/G2 antibodies were characterized by direct ELISA format, using E2/G2 as a capture and 2,4-D-HRP conjugate as a detection reagent. Immunoaffinity chromatography columns were prepared and characterized using a modified version of a protocol described by Rolčík *et al*. (2002)[39]. Briefly, the IAG was prepared by coupling of the monoclonal antibodies (25 mg) with 1 ml of Affi-Gel 10 (Bio-Rad, USA) in 5 ml cartridges. Subsequently, the IAG was regenerated with a cycle of 3 ml portions of H_2_O-MeOH-H_2_O, re-conditioned by 3 ml PBS (50mM NaH_2_PO_4_, 15mM NaCl, pH 7.2) and finally used for sample enrichment. After methanolic elution, the samples were evaporated to dryness prior to UHPLC-MS/MS analysis. The process efficiency of the two-step isolation method was examined using crude plant extracts (15 mg fresh weight) in quadruplicates, which were spiked with known concentrations of 2,4-D (0.5, 5 and 50 pmol) and the recoveries of analyte were calculated.

### UHPLC-MS/MS conditions

For quantitative analysis of 2,4-D and its metabolites/analogs, ACQUITY UPLC^®^ I-Class System (Waters, USA) combined with Xevo^TM^ TQ-S MS (Waters, UK) were used. The samples were injected onto a reversed-phase column (Acquity UPLC^®^ BEH C18, 1.7μm, 2.1x50 mm; temperature 40°C) and eluted with a linear gradient (0–7 min, 35–65% B; 7–8 min, 100% B; 8–10 min, 35% B) of aqueous 0.1% formic acid (A) and 0.1% formic acid in methanol (B) at a flow-rate of 0.25 ml min^-1^. Quantification was obtained by multiple reaction monitoring (MRM) mode of precursor ion ([M-H]^–^) and the appropriate product ion. The MRM transitions and the MS settings are listed in S3 Table. The calibration curves ranging from 50 fmol to 250 pmol were constructed by serial dilutions of the authentic standards and the known concentration of the appropriate internal labeled standards. The concentrations of the 2,4-D metabolites were calculated by isotopic dilution method according to a known quantity of an internal standard added during the extraction step.

### Quantification of IAA and its amide-linked conjugates

Extraction and purification of auxin metabolites were done as described previously by Novák *et al*. (2012)[8] with minor modifications. Frozen samples were homogenized using a MixerMill (Retsch GmbH, Haan, Germany) and extracted in 1 ml 50 mM sodium phosphate buffer (pH 7.0) containing 1% sodium diethyldithiocarbamate, ^13^C-labeled internal standards. The samples were purified on Oasis HLB columns (30 mg, Waters Corp., Milford, USA), eluates were evaporated to dryness and dissolved in 20 μl of mobile phase prior to mass analysis using an ACQUITY UPLC^®^ I-Class System and Xevo^TM^ TQ-S MS.

## Results

### 2,4-D metabolite inhibits primary root growth through an auxin-mediated signaling pathway

**Fig 1:**
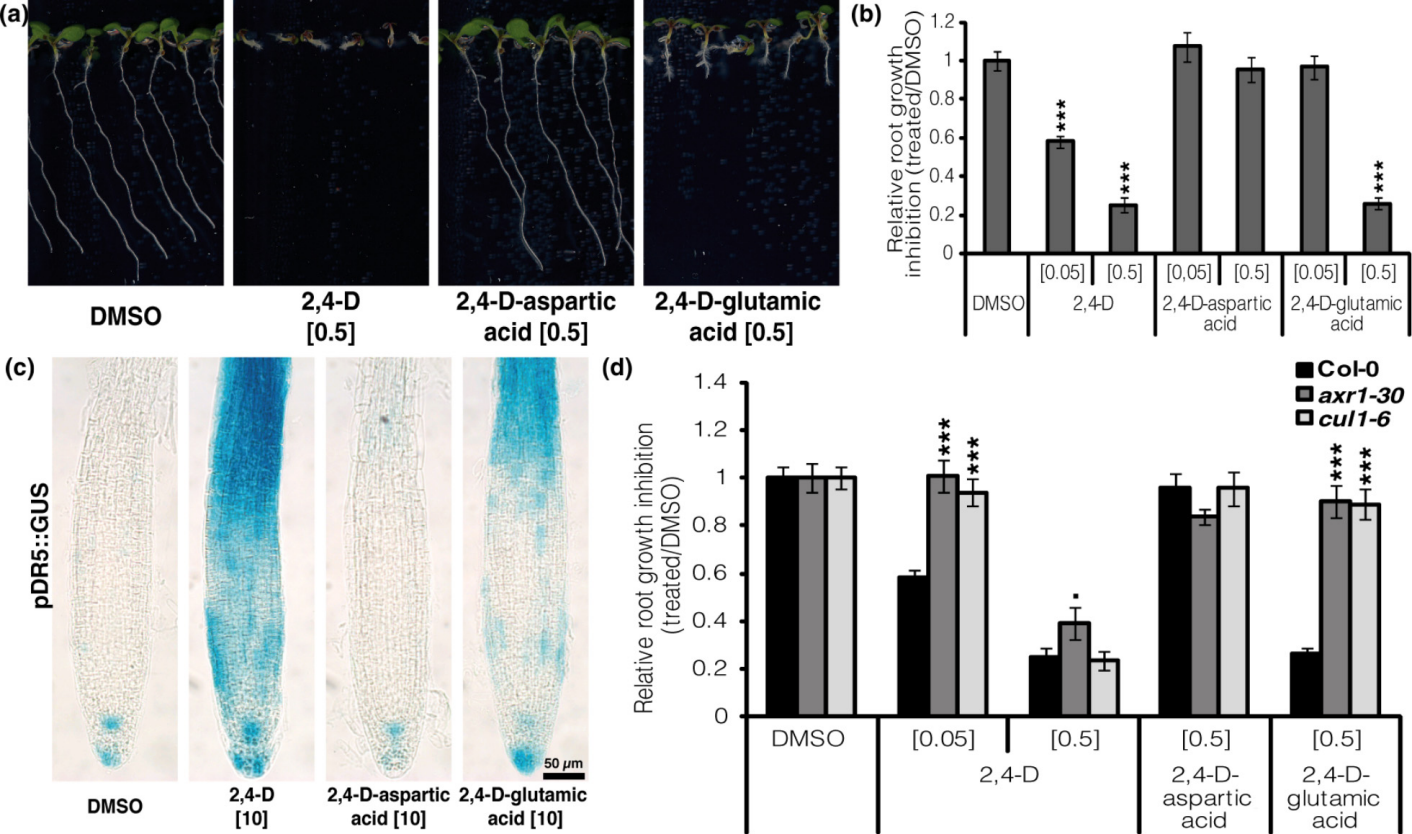
2,4-D and 2,4-D metabolites act on *Arabidopsis thaliana* through auxin signaling pathway. (A) Col-0 seedlings were grown for 7 days on 2,4-D, 2,4-D-aspartic acid and 2,4-D-glutamic acid. DMSO was used as control. 2,4-D and 2,4-D-glutamic acid induced a clear reduction of primary root growth at 0.5 μM. (B) Relative root growth inhibition quantification. (C) Expression of pDR5::GUS in the primary root meristem after DMSO, 2,4-D, 2,4-D-aspartic acid and 2,4-D-glutamic acid treatments (5 day-old seedlings, 5 h treatment, 10 μM). Similarly to 2,4-D, 2,4-D-glutamic acid strongly induced the expression of pDR5::GUS in the primary root meristem. 2,4-D-aspartic acid slightly induced expression of pDR5::GUS in the elongation zone of the primary root. (D) Col-0, *axr1-30* and *cul1-6* seedlings were grown for 7 days on 2,4-D, 2,4-D-aspartic acid and 2,4-D-glutamic acid. DMSO was used as control. *axr1-30* and *cul1-6* lines showed significantly less sensitivity to the primary root growth inhibition observed in Col-0 with 2,4-D at 0.05 μM and 2,4-D-glutamic acid at 0.5 μM. In (B) and (D), values represent n>30 from three independent experiments. Statistical analysis was performed using ANOVA & comparison of means (Tukey’s test) to relative root growth inhibition of DMSO (B) and Col-0 (D). No asterisks indicate p-value < 0.1; *: p-value < 0.05; **: p-value < 0.01; ***: p-value < 0.001.

Initially we tested the potential bio-activity of the 2,4-D amide-linked conjugates. The physiological activities of 2,4-D, 2,4-D-Glu and 2,4-D-Asp were tested in wild-type *Arabidopsis* seedlings. The seedlings grown in the presence of 2,4-D and to a lesser extent 2,4-D-Glu, displayed dose-dependent inhibition of primary root development and induction of lateral root formation (Fig 1A and 1B). At comparable concentrations, no inhibitory effect on plant growth was observed after 2,4-D-Asp treatment. Overall, these data demonstrate that 2,4-D-Glu displays a potential activity on seedling growth but with a lower potency than 2,4-D. This activity might be directly attributed to 2,4-D-Glu or to a degradation product to free 2,4-D.

2,4-D is known to act through the TIR1/AFB auxin-mediated signaling pathway[22]. In order to understand the mode of action of 2,4-D-Glu, the activities of 2,4-D, 2,4-D-Glu, and 2,4-D-Asp were tested on mutants deficient in the auxin-mediated signaling pathway—*axr1-30* [36] and *cul1-6* [40]. In sharp contrast to the Col-0 wild-type lines, the *axr1-30* and *cul1-6* mutants displayed decreased sensitivity to 2,4-D-Glu (0.5 μM). A similar decrease in sensitivity of *axr1-30* and *cul1-6* mutants was observed in plants treated with low concentration of 2,4-D (0.05 μM). However, at high concentration of 2,4-D (0.5 μM), a strong root growth inhibition occurred equally in the wild-type and the auxin-signaling deficient mutants, suggesting that at 0.5 μM, 2,4-D exhibits a toxic activity (Fig 1D). These data support the idea that the nuclear auxin signaling machinery mediates 2,4-D action as shown before, but also show that this machinery mediates 2,4-D-Glu derived activities as well. To further investigate the mode of action of 2,4-D-Glu, 2,4-D and both 2,4-D-amino acid conjugates were assessed for their ability to affect the expression of pDR5::GUS, a synthetic auxin-responsive marker line commonly used to image auxin response [37] (Fig 1C). Similarly to 2,4-D, the plants treated with 2,4-D-Glu showed an increased pDR5::GUS expression in the primary root tip after 5 hours. This indicates that 2,4-D-Glu is able to directly or indirectly induce an auxin response. Interestingly, even though no 2,4-D-Asp-induced phenotypes were observed in seedlings, 2,4-D-Asp slightly induced pDR5::GUS expression in the root elongation zone (Fig 1C), suggesting a very low activity.

### Class-specific isolation of 2,4-D and its amino acid conjugates

In order to investigate the catabolic/conversion products of 2,4-D and 2,4-D-conjugates, we first established a method to pre-concentrate 2,4-D related compounds from plant tissues. The metabolites of nine-day-old *Arabidopsis* seedlings grown on media supplemented by the respective compounds were extracted. The extracts were subsequently enriched by a solid-phase extraction (SPE) to increase method selectivity (S1 Fig) and immunoaffinity purification to selectively capture trace amounts of 2,4-D and its metabolites from complex plant matrices using the monoclonal antibodies E2/G2 [38]. The IAC is a powerful isolation tool to reduce large proportions of potentially interfering substances and also to increase the sensitivity of the subsequent LC-MS/MS analysis, since interferences by the sample matrix can be reduced, resulting in an increased signal-to-noise ratios. According to the cross-reactivity study, the E2/G2 antibodies preferably recognized compounds with the 2,4-dichlorophenoxyacetic moiety (S4 Table). Moreover, the capacity of the immunoaffinity gel (IAG) is an important parameter, which is defined as the maximum amount of the analyte that can be pre-concentrated by a given volume of immunosorbent [41]. Thus, to test our column capacities, 2,4-D standards ranging from 1 pmol to 1 nmol were applied to 0.5 ml of the gel and the recovery was determined by UHPLC-MS/MS. The IAG capacity was estimated to be around 200 pmol ml^-1^. Up to this concentration of 2,4-D recovery was still higher than 50% (S2 Fig). Beyond this limit, the immunoextraction recovery declined rapidly. Therefore, the yields of 2,4-D metabolites and selected structural analogs were also tested in a wide concentration range from 1 to 300 pmol (Table 1; S4 Table). The results showed good affinity of the E2/G2 antibodies to specifically bind the 2,4-D-amino acid conjugates and to a lesser extent some structural analogs (2,4-dichlorophenoxybutyric acid, 2-methyl-4-dichlorophenoxybutyric acid and 2,4,5-trichlorophenoxyacetic acid). After SPE and class-specific IAC employing the monoclonal E2/G2 antibodies, the total recoveries for 2,4-D, 2,4-D-Glu and 2,4-D-Asp were 66 ± 17%, 28 ± 8% and 16 ± 6% (n = 12), respectively (S5 Table). This result indicates that the method is appropriate for the routine isolation of 2,4-D and its conjugates from plant tissue.

**Table 1:** E2/G2 monoclonal antibodies and immunoaffinity gel characteristics.

**Compound**	**CR (%)**	**Recovery (%)**					
		**1 pmol**	**5 pmol**	**10 pmol**	**50 pmol**	**100 pmol**	**300 pmol**
**2,4–D**	100.0	91.5 ± 7.9	75.3 ± 4.3	69.4 ± 4.9	59.2 ± 2.3	47.6 ± 7.9	32.6 ± 5.8
**2,4–D–Asp**	46.4	48.3 ± 9.6	14.7 ± 2.4	9.3 ± 1.4	7.2 ± 0.2	1.6 ± 0.3	0.8 ± 0.1
**2,4–D–Glu**	60.0	36.1 ± 6.6	31.8 ± 0.6	19.9 ± 1.5	18.0 ± 1.4	6.8 ± 1.2	4.9 ± 0.1

Percentage of cross reactivity (CR) was calculated using the equation: CR(%) = IC50(2,4-D)/IC50(cross reactant)x100, where IC50 is the concentration of a competitor (cross reactant) resulting in 50% reduction of alkaline phosphatase conjugate binding in direct ELISA system. Mixtures of 2,4-D structural analogs with concentrations ranging from 1 to 300 pmol were applied onto 0.5 ml of IAG, each spiking level was then determined by UHPLC-MS/MS, compared with the concentration of appropriate standard solution and the recoveries were calculated (values are means ± SD, n = 3).

### 2,4-D metabolite profiling by UHPLC-MS/MS

**Fig 2:**
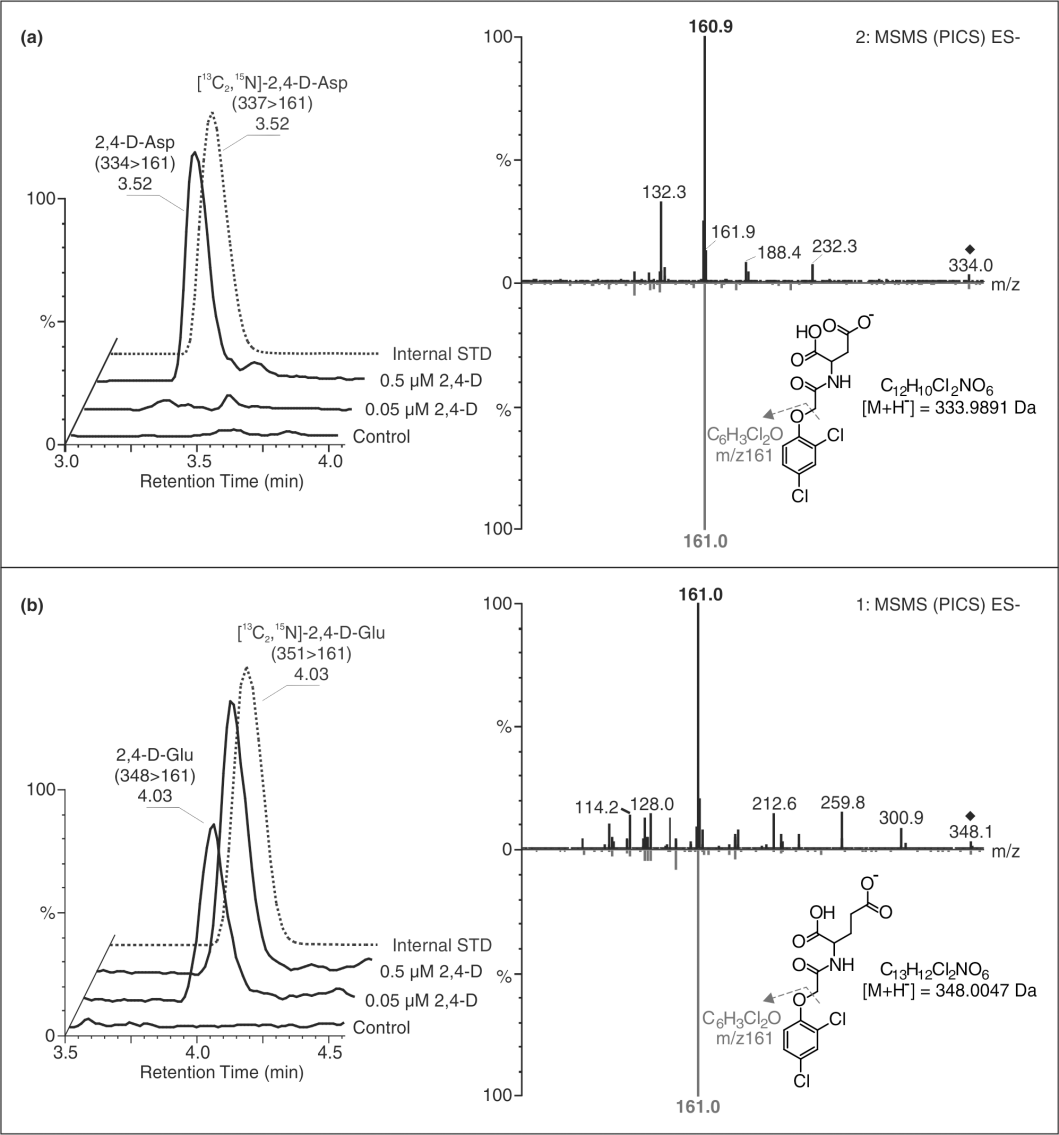
Confirmation of presence of 2,4-D-amino acid conjugates in *Arabidopsis* plant tissues treated with 2,4-D. *Left*: MRM chromatograms of 2,4-D-Asp (A) and 2,4-D-Glu (B) show their identification in extracts of treated (0.05 μM and 0.5 μM of 2,4-D) and untreated (control) plants based on retention time and co-elution with appropriate internal standards (STD, grey color). *Right*: Product Ion Confirmation Spectra (PICS) of endogenous 2,4-D-Asp (a) and 2,4-D-Glu (b) (black color) in comparison with the standards synthesized (grey color) validates the presence of 2,4-D-amino acid conjugates in plant tissue samples purified by IAC.

The newly developed two-step purification procedure has been linked to ultra-high performance liquid chromatography–tandem mass spectrometry (UHPLC-MS/MS). During the MS-based analysis, a mixture of eight 2,4-D metabolites/analogs could be baseline separated over 7.0 min (as shown in S3 Fig). The chromatographic stability was tested in detail by 10 consecutive measurements (S3 Table) and coefficients of variation for the retention times were found to range between 0.08% and 0.30% relative standard deviation (RSD), showing high levels of consistency during the chromatographic separations. 2,4-D and its amino acid conjugates were detected in negative-ion, multi reaction monitoring (MRM) mode with a strong signal from the deprotonated molecule [M–H]^−^and high-intensity fragment of 2,4-dichlorophenoxy moiety (m/z 161) (Fig 2). Furthermore, we have used rapid switching between two modes of operation, MRM and product ion confirmation spectrum (PICS) modes for a simultaneous quantification and a structural confirmation by sensitive MS/MS full scan [42]. After optimizing the MS/MS conditions, correlation coefficients between 0.9985 and 0.9995 were calculated in the linear dynamic ranges from the lower limits of detection close to 20 fmol using repeated injection of all investigated 2,4-D metabolites (S3 Table).

We also validated both parts of the analytical method together (purification and quantification) using a spiking experiment of crude plant extracts (15 mg fresh weight of *Arabidopsis* seedlings). The precision and accuracy of the whole procedure is shown in S5 Table. For 0.5 pmol (low), 5 pmol (medium) and 50 pmol (high) concentrations of 2,4-D metabolites, the mean precision was 6.0% RSD (in the range 1.4%–10.6%), and the mean accuracy was 5.9% bias (in the range -3.6% to 18.3%). Overall, these results confirm that our new method is a powerful, precise and accurate tool for the target profiling of 2,4-D metabolites.

### *In vivo* metabolism of 2,4-D and its amino acid conjugates

**Fig 3:**
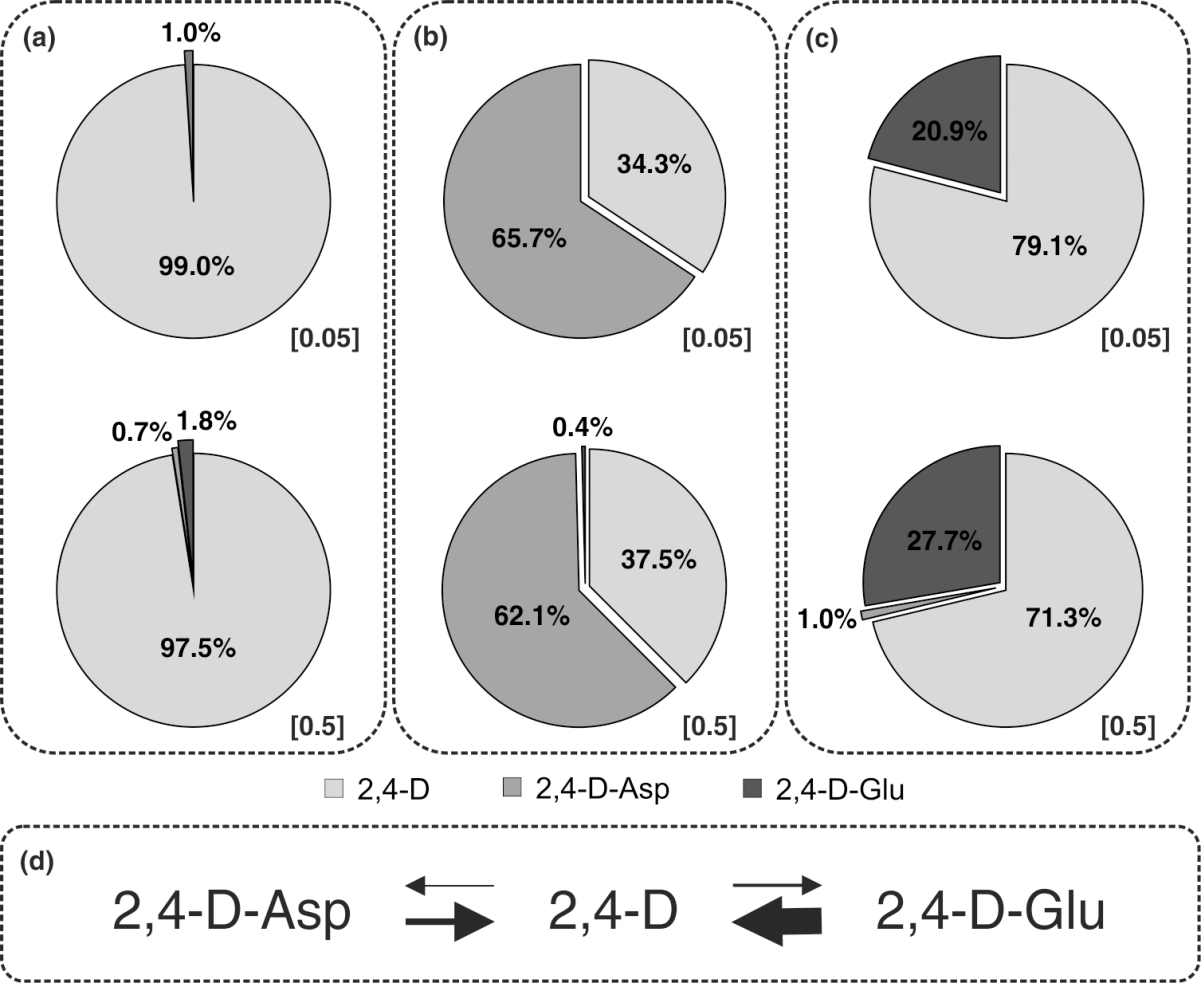
Distribution (%) of 2,4-D and its metabolites in Arabidopsis seedlings. Plant tissues were grown on media supplemented with two concentrations (0.05 μM and 0.5 μM) of 2,4-D (A), 2,4-D-Asp (B) and 2,4-D-Glu (C), and metabolite distribution in each treatment was calculated from the levels (pmol g-1 FW) detected by UHPLC-MS/MS. In (D), 2,4-D homeostasis and different biosynthetic rates from/to free 2,4-D are indicated (the line thickness illustrates a predicted conversion rate of 2,4-D and its amino acid conjugates).

Having established a specific immunoaffinity-based and sensitive MS-based approach, we studied the 2,4-D metabolism *in vivo*. The extracts purified using a SPE column (Oasis^®^ MAX, 1 ml/30 mg, Waters) followed by immunoaffinity chromatography were injected onto a reverse-phase chromatographic column for quantification of the 2,4-D-amino acid conjugates and confirmation of their presence in the plant tissues grown on media supplemented with 2,4-D and 2,4-D-conjugates (Fig 2). In 2,4-D treated seedlings, 2,4-D-Glu and 2,4-D-Asp were detected at 100-fold lower concentrations in comparison with 2,4-D levels (Table 2), confirming the high metabolic stability of 2,4-D. This also clearly demonstrates the relationship between 2,4-D uptake and formation of amide-linked conjugates in *Arabidopsis* seedlings (Fig 3A).

**Table 2:** Levels of 2,4-D and its metabolites in 1 g of extracted *Arabidopsis* tissue.

	2,4-D	2,4-D-Asp	2,4-D-Glu
		0.05 μM	
**2,4-D**	761 ± 208	40 ± 10	133 ± 31
**2,4-D-Asp**	n.d.	77 ± 10	n.d.
**2,4-D-Glu**	8 ± 1	n.d.	35 ± 12
		0.5 μM	
**2,4-D**	3258 ± 85	519 ± 97	1295 ± 296
**2,4-D-Asp**	24 ± 5	858 ± 156	19 ± 1
**2,4-D-Glu**	59 ± 3	5 ± 2	504 ± 113

The concentration for all metabolites is in pmol g^-1^ FW. For 0.05 μM treatment, the amount of 2,4-D-Asp was not detected (n.d.). Samples were analyzed in four independent biological replicates, and error bars represent the SD.

Next we treated 9-day-old seedlings with 2,4-D amino acid conjugates and detected free 2,4-D levels (Table 2). This observation is in good agreement with previous studies [27,28] showing that metabolism of 2,4-D-conjugates results in conversion into the free 2,4-D molecule. This hydrolysis did not occur in the growth media by itself, indicating that it depends on inherent enzymatic activity (S4 Fig). Interestingly, a higher proportion of 2,4-D was observed in the 2,4-D-Glu-treated samples (more than 70% converted as shown in Fig 3C), compared to samples treated with 2,4-D-Asp (only about 35%, Fig 3B), suggesting that both amide-linked conjugate forms are degraded with different metabolic rates and/or catabolic pathways. Alternatively, the cellular uptake of the compounds may differ, because exogenous application of 2,4-D-Glu led to higher compound accumulation in plant tissues as compared to 2,4-D-Asp.

Remarkably, the hydrolysis efficiency was independent of the initial concentration of 2,4-D-amino acid conjugates supplemented in the growth medium (Fig 3B and 3C), showing approximately the same proportion of 2,4-D in concentration ranges of 0.05 μM and 0.5 μM. Notably, 2,4-D-Glu was determined as the minor metabolite in the plant tissues previously treated with high concentration of 2,4-D-Asp (0.5 μM), indicating that formed 2,4-D is rapidly secondarily metabolized. Similarly, minor amounts of 2,4-D-Asp were detected in plants treated with 0.5 μM 2,4-D-Glu. These findings arguably favor the possibility that the divergent auxin-like activities of both 2,4-D amide-linked conjugates are mediated by different cellular uptake and metabolic conversion to free 2,4-D (Fig 3D).

### Endogenous and synthetic auxin conjugates show distinct metabolism

**Fig 4:**
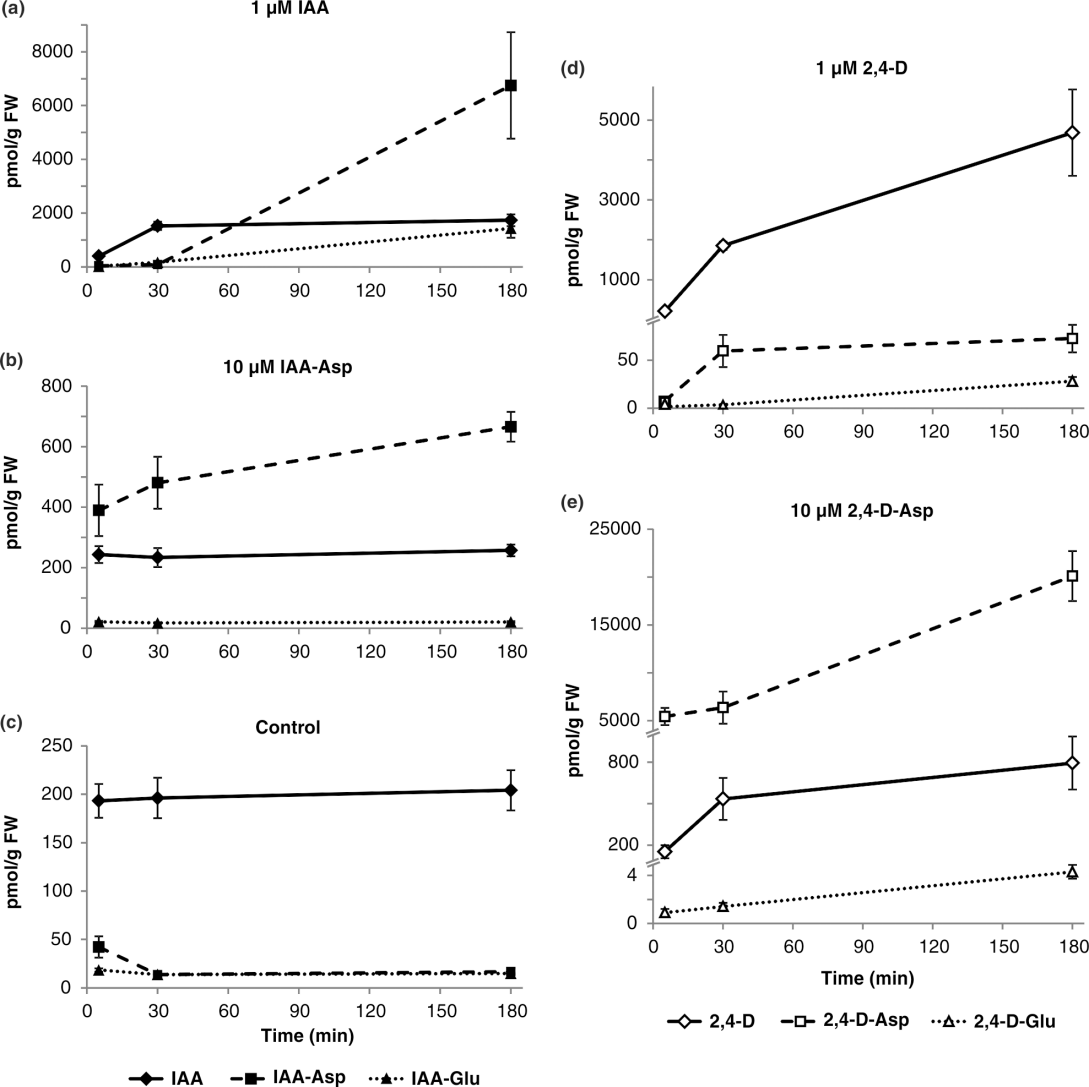
Short-term metabolization of IAA, 2,4-D and their conjugates with aspartate in *Arabidopsis*. Extracts from seven-day-old Col-0 seedlings pre-incubated for 5 min, 30 min and 3 hours with the indicated compounds (A, 1 μM of IAA; B, 10 μM of IAA-Asp; D, 1 μM of 2,4-D; E, 10 μM of 2,4-D-Asp) were analyzed by UHPLC-MS/MS and compared with a endogenous auxin levels in the control untreated seedlings (C). The concentration for all metabolites is in pmol g^-1^ FW. Samples were analyzed in four independent biological replicates, and error bars represent the SD.

Our data reveals that 2,4-D-Asp and 2,4-D-Glu show expressive hydrolysis to 2,4-D, affecting plant growth and development. This is an unexpected finding, because endogenous conjugation of IAA to IAA-Glu and IAA-Asp has been suggested to be non-reversible (reviewed in Ludwig-Mueller, 2011). Accordingly, exogenous application of IAA-Asp does not itself affect plant development [43]. To compare metabolism of synthetic and endogenous IAA conjugates we applied the endogenous compounds IAA and IAA-Asp as well as synthetic 2,4-D and 2,4-D-Asp for 5, 30 and 180 minutes (Fig 4). The initial accumulation of IAA and 2,4-D in plant tissues was comparable during the first 30 minutes. However, while IAA concentration saturated, the 2,4-D accumulation further increased (Fig 4D). IAA saturation correlated with increased abundance in its amide-linked conjugates, such as IAA-Asp and IAA-Glu (Fig 4A), suggesting that auxin conjugation evokes the distinct accumulation rates of IAA and 2,4-D exceeding 30 minutes.

Next we investigated tissue accumulation and metabolism of IAA-Asp and 2,4-D-Asp. In contrast to IAA and 2,4-D, we detected a similar accumulation of both IAA-Asp and 2,4-D-Asp in plant tissues (Fig 4B and 4E). Compared to IAA-Asp, the uptake of 2,4-D-Asp was higher in plant tissues. IAA-Asp treated samples showed only slightly increased endogenous levels of IAA as compared to the control treated samples (Fig 4B). However, 2,4D-Asp treated samples showed much higher conversion to 2,4-D (Fig 4E), suggesting that not only the metabolism rates of 2,4-D and IAA, but also 2,4-D-Asp and IAA-Asp are distinct. Whereas 2,4-D appears more stable compared to IAA, we propose that 2,4-D amino acid conjugates, such as 2,4-D-Asp, is less stable *in vivo* than IAA-Asp. To confirm our findings, we have tested the short-term stability of 2,4-D and its amino acid conjugates in light and/or in the medium without *Arabidopsis* seedlings (S5 Fig). These results showed no significant change in the exogenous levels of conjugated 2,4-D in 5–180 min after treatment. Accordingly, 2,4-D amide-linked conjugates could be used as a vehicle to intracellularly release 2,4-D.

Our data indicates that amino acid conjugation to synthetic auxins could lead to distinct metabolic turnover as compared to endogenous compounds. This insight could be used to engineer sophisticated auxin compounds to dissect metabolic processes in planta and to establish novel classes of possibly selective herbicides getting released only intracellularly.

## Discussion

In plant hormone research, application of synthetic auxin such as 2,4-D has already unraveled the activity of endogenous IAA in specific developmental events [44]. By a multidisciplinary approach combining organic synthesis, chemical biology and quantitative analysis, we deepened the understanding of 2,4-D mode of action and propose a new method using synthetic analogs to monitor auxin action linked to auxin metabolism.

Having established an effective procedure, we first profiled *in vivo* conversion of 2,4-D into conjugated metabolites (Table 2). In plant callus tissues treated with radioactive 2,4-D, amide-linked conjugates have been previously reported as major identified metabolites [45]. 2,4-D, often used for its high metabolic stability, showed under our incubation conditions conjugation with aspartic and glutamic acids–both metabolites were present in very low concentration of only 1–2% of all 2,4-D metabolite contents (Fig 3A), confirming a previous report where they represented only 3% of the conjugated pool throughout the whole *Arabidopsis* plant [46]. The distribution of 2,4-D-amino acid conjugates is also in agreement with previously reported results for treated corn plants (*Zea mays* L.) in which sugar conjugation of 2,4-D was predominant [23]. Interestingly, this result shows the link to auxin metabolism, describing ester-linked sugars as the major conjugate form of IAA in maize kernels [47]. Similarly, in pedicel explants of tobacco, another synthetic auxin, 1-naphthaleneacetic acid (1-NAA), was transformed firstly to a glucose ester and secondly to aspartic acid amide (NAA-Asp) [48]. As a consequence, we propose that 2,4-D homeostasis is controlled by a constant synthesis and breakdown of its derivatives. Our data further indicate that amide-linked 2,4-D conjugates could be formed and/or accumulated with different biosynthetic rates. Indeed, compared to quantitative measurements of IAA-Asp and IAA-Glu in wild-type *Arabidopsis* lines [8,49], we found the opposite ratio of 2,4-D-Glu to 2,4-D-Asp levels (Table 2). IAA conjugates are generally postulated as being intermediate substrates for the IAA transport machinery, or as IAA storage forms, or as a protection from enzymatic destruction leading to the homeostatic control of IAA [50]. Conversely, in *Arabidopsis*, the most abundant amide-linked IAA conjugates, IAA-Asp and IAA-Glu, are not measurably hydrolyzed to free IAA and are thought to most likely be intermediates in IAA catabolism [6]. It is therefore very difficult to monitor the IAA conjugation/deconjugation ratio *in vivo*. The production and quantification of traceable synthetic auxin and/or auxin conjugates will undoubtedly pave the way to a better understanding of auxin metabolism *in vivo* and could lead to precise *in vivo* characterization of conjugating enzyme activities.

Secondly, to investigate the activity of 2,4-D metabolites, we have successfully synthesized 2,4-D conjugated with L-glutamic and L-aspartic acid (S1 Table, S2 Table). By conducting a chemical biology approach we could show that treatment with 2,4-D conjugates induced a growth phenotype in *Arabidopsis* seedlings, dependent on the SCF hormone signaling pathway (Fig 1). The increase of auxin-responsive reporter expression after 5 hours suggests a fast uptake of the chemical by the plant. Notably, the 2,4-D conjugated with aspartic acid is less potent than 2,4-D-Glu and they are both less active than 2,4-D itself. In previous studies, 2,4-D showed a growth stimulation effect at the optimum concentration of 10^−6^ M, while twenty synthetic amide-linked 2,4-D conjugates also displayed the ability to enhance elongation of *Avena* coleoptile sections but at higher concentrations (10^−5^–10^−6^ M) [29,51]. In *Arabidopsis*, seven gene members of the auxin-inducible GH3 family of amido synthetases are able to catalyze the synthesis of IAA amide conjugates [17,52]. As summarized in Westfall *et al*. (2010) [53], most IAA-specific GH3 proteins conjugate IAA to Asp and/or Glu. Similarly in grapevine, modelling of 1-NAA into the active site of GH3-1 suggests that 1-NAA is likely to be a poor substrate for this enzyme [54]. As our data suggest a low conjugation rate of 2,4-D into 2,4-D-Asp and 2,4-D-Glu, it would be relevant to test the capacity of 2,4-D to be a good substrate for the GH3 protein family and compare it to the data already collected for IAA and 1-NAA. Overall, these data offer two possibilities with regard to conjugate activity: (i) the amino acid conjugates of 2,4-D could be the physiologically active forms (as postulated in 51), (ii) only 2,4-D is the active molecule, if it could be formed as a product of the metabolism of the amide-linked 2,4-D conjugates. However, one should not forget to consider the possibility of different uptake capacity relative to the molecule [55].

To further understand the physiological effects of 2,4-D metabolites, we have applied novel analytical approaches to isolate and profile the catabolic/conversion products (S1 Fig). As the concentrations of synthetic auxin metabolites investigated in plant tissues are usually extremely low (pmol.g^-1^ fresh weight), their determination required sample clean-up steps prior to analyte detection. Therefore, the combination of ion-exchange and class-specific sorbents was used for sample enrichment. The Oasis^®^ MAX sorbent based on both reverse-phase and anion-exchange has excellent preconditions to separate acidic analytes from neutral compounds [56]. Using previously prepared and characterized E2/G2 monoclonal antibodies [38] (Table 1), we describe here the procedure for efficient sample extraction and purification of 2,4-D and its conjugated metabolites (S1 Fig). All validation parameters (S4 Table; S5 Table), the IAG capacity and the process efficiency, were consistent with phytohormonal IAC-based methods published recently [39,57,58]. We have developed and optimized MS-based method for multiplex confirmation and quantification of 2,4-D metabolites (Fig 2). The MS detection using MRM and PICS modes amplifies the accuracy and precision of trace component analysis in a complex plant matrix. In agreement with our findings (S3 Table; S5 Table), the overall sensitivity of our analytical method enables analysis of 2,4-D and its amino acid conjugates from minute amounts of plant tissue. Moreover, we have shown the immunoaffinity chromatography gel to be highly specific for 2,4-D, certain 2,4-D metabolites, and other structurally related compounds, making it highly useful for purifying 2,4-D analogs in complex sample extracts (S4 Table). Our approach provides very efficient confirmatory tools that can discriminate auxinic herbicides at ultra-trace levels for reliable and sensitive detection to help ensure food safety.

In auxin metabolism, conjugation is generally considered to be either a reversible or irreversible process of degradation leading to attenuation of auxin activity. Previous studies in soybean cotyledon callus tissue demonstrated that 2,4-D-Glu converts to free 2,4-D and other conjugates [27,29]. Our MS-based data show that 2,4-D-Asp is also hydrolyzed to free 2,4-D as well as glutamic acid conjugate (Fig 3B and 3C). Interestingly, in *Arabidopsis* plants treated with the amino acid conjugates, the level of free 2,4-D was found to be 1.7-fold higher and 3.2-fold lower compared to the concentration of 2,4-D-Glu and 2,4-D-Asp, respectively (Table 2). Based on these results, we conclude that the accumulation of free 2,4-D is connected with the divergent auxin-like activities of both amino acid conjugates. 2,4-D glutamic acid, similarly to 2,4-D, inhibits primary root growth and also acts through an auxin-mediated signaling pathway. Altogether, our quantitative data suggest that 2,4-D-Glu is strongly hydrolyzed to free 2,4-D (<70% of all 2,4-D metabolite levels; Fig 3C) and physiological activity is formed as a product of the amide conjugate metabolism. On the other hand, the lower concentrations of free 2,4-D (>40% of total content; Fig 3B) in the seedlings grown on media supplemented 2,4-D-Asp correspond with no reduction of primary root length and very low activity in the auxin response. These data are consistent with the finding by early studies of auxin metabolism that 1-NAA conjugated with aspartic acid is hydrolyzed very slowly and does not affect the growth of tobacco crown-gall tissues [59]. Furthermore, 1-NAA-Asp acts as an auxin only after hydrolysis to 1-NAA [60]. LeClere *et al*. (2002)[61] tested the ability of IAA-amino acid conjugates to inhibit *Arabidopsis* seedling root growth and compared the *in vitro* enzymatic activity of four *Arabidopsis* IAA-amino acid hydrolases (ILR1, IAR3, ILL1 and ILL2). In accordance with our findings, the aspartic acid conjugate was inactive in root inhibition bioassays and very slightly cleaved by ILR1 and ILL2. However, compared to IAA-Asp, IAA-Glu showed a two-fold increase in substrate specificity of *Arabidopsis* amidohydrolases and a slight activity on root elongation inhibition [61]. According to our finding, we suggest that 2,4-D-Glu is also efficiently hydrolyzed *in vivo* by the amidohydrolases. Taken together, our data strongly indicate that 2,4-D-Asp and 2,4-D-Glu are reversible forms of 2,4-D homeostasis that can be converted to free 2,4-D with different biosynthetic rates (Fig 3D).

Overall, our results demonstrate that 2,4-D is conjugated *in vivo* and that 2,4-D conjugates can be hydrolyzed back to the active form of 2,4-D. Furthermore, free 2,4-D is active on the TIR1/AFB-mediated auxin signaling pathway and not its conjugated forms. As 2,4-D is a poor substrate for ABP1, a discussed potential auxin receptor [62,63], TIR1/AFB and the related auxin signaling pathway have been shown to be the primary signaling machinery targeted by 2,4-D. Moreover and based on structural evidences, 2,4-D-Asp or 2,4-D-Glu would not be able bind to the TIR1-Aux/IAA co-receptor complex. This study paves the way to allow for new experiments linking the nuclear auxin signaling pathway and the regulation of auxin conjugation by the use of traceable synthetic auxin. The hereby technology established to synthetize and quantify *in vivo* 2,4-D forms will lead the way to novel technologies such as the production of 2,4-D-labeled molecules and *in vivo* detection, which would be a specific read-out of TIR1/AFB signaling processes. Expressing inducible amidohydrolases or conjugation enzymes could modulate the cellular 2,4-D amount in a tissue or in a time-controlled manner. This is of fundamental importance to better understand the auxin effect on plant architecture and also from a more applied point of view. For example, some commercially valuable plants could be genetically manipulated to increase their GH3 level and thus acquire a resistance to the herbicide effect of 2,4-D. Overall, the 2,4-D and 2,4-D metabolites represent fantastic tools for biotechnology approaches.

## Supporting Information

S1 Fig Scheme of a two-step purification protocol for isolation of 2,4-D and its metabolites/structural analogues. (PDF)

S2 Fig Capacity of the immunoaffinity gel (IAG) with immobilized E2/G2 antibodies. (PDF)

S3 Fig Chromatographic separation of 2,4-D and its metabolites/structural analogs by UHPLC-(ESI–)-MS/MS. (PDF)

S4 Fig One-week stability of 2,4-D-amino acid conjugates in growth media. (PDF)

S5 Fig Stability of 2,4-D, 2,4-D-Asp and 2,4-D-Glu in short-term chemical treatment. (PDF)

S1 Table Preparation scheme and structural characteristics of 2,4-D-Asp. (PDF)

S2 Table Preparation scheme and structural characteristics of 2,4-D-Glu. (PDF)

S3 Table Optimized UHPLC-(ESI–)-MS/MS parameters. (PDF)

S4 Table Characterization of monoclonal E2/G2 antibodies and immunoaffinity gel by 2,4-D structural analogues. (PDF)

S5 Table Process efficiency and method validation for the two-step purification procedure. (PDF)
