# Supplementary material for: 2,4-D and IAA Amino Acid Conjugates Show Distinct Metabolism in Arabidopsis
Source: PLoS One. 2016 Jul 19;11(7):e0159269. doi: 10.1371/journal.pone.0159269 (PMC4951038; doi:10.1371/journal.pone.0159269)
Supplement: S1 Fig — (PDF) [file pone.0159269.s001.pdf]

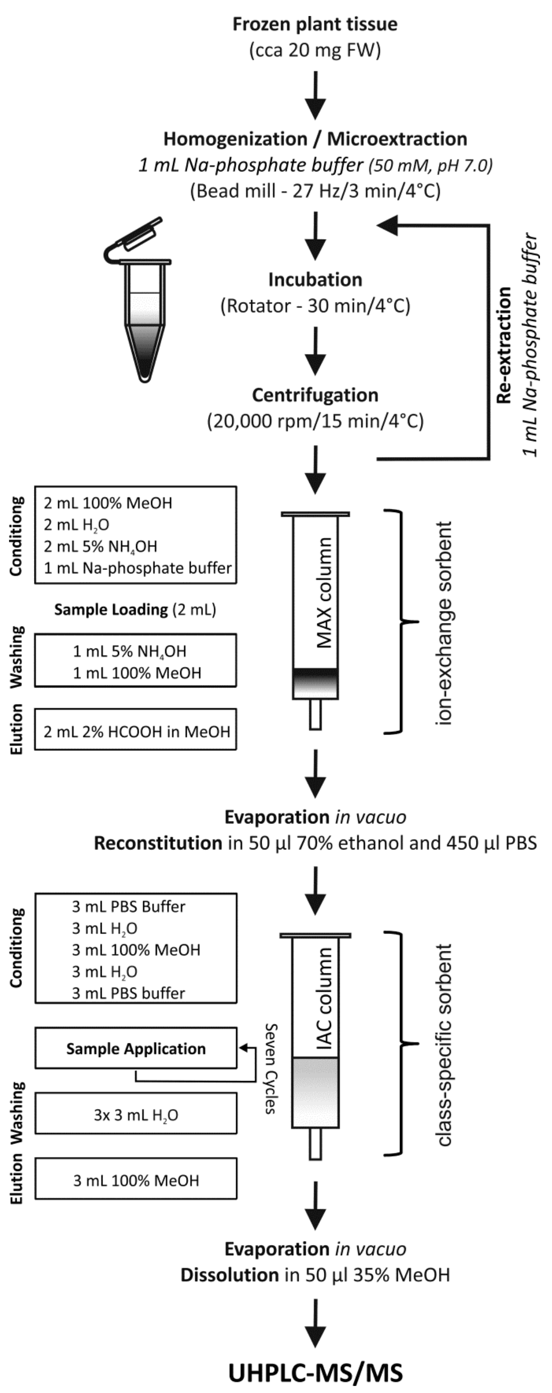

S1 Fig. Scheme of sample preparation process for 2,4-D and its metabolite/structural analogue determination using the ion-exchange and class-specific sorbents in two-step purification protocol. Plant material (20 mg FW) was extracted using 50mM Na-phosphate buffer (pH 7.0) with stable isotope-labelled standards. The extracts were purified using the mixed-mode Oasis® MAX cartridges (1 ml/30 mg). The eluates were evaporated, then reconstructed and repeatedly applied onto immunoaffinity chromatography (IAC) columns. The immunoaffinity gel was subsequently washed and the bound 2,4-D metabolites were eluted by 3ml 100% methanol. All obtained fractions were evaporated to dryness, stored at -20°C until UHPLC-ESI(–)-MS/MS analysis (10 µL of sample injected).
