# Supplementary material for: 2,4-D and IAA Amino Acid Conjugates Show Distinct Metabolism in Arabidopsis
Source: PLoS One. 2016 Jul 19;11(7):e0159269. doi: 10.1371/journal.pone.0159269 (PMC4951038; doi:10.1371/journal.pone.0159269)
Supplement: S2 Fig — (PDF) [file pone.0159269.s002.pdf]

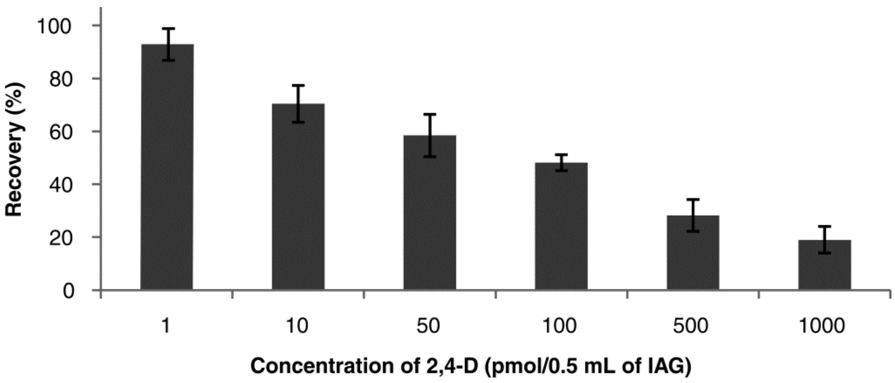

S2 Fig. Capacity of the immunoaffinity gel (IAG) with immobilized E2/G2 antibodies presented as analyte recoveries observed in tests with 0.05–1 nmol of 2,4-D. Solution of 2,4-D with concentrations ranging from 1 to 1000 pmol in methanol:PBS buffer (5:95, v/v) were applied onto 0.5 ml of IAG. The eluates were evaporated to dryness, dissolved in 50 µL of 35% methanol and analysed by UHPLC-ESI(–)-MS/MS (10 µL of sample injected). Each spiking level was then determined, compared with the concentration of appropriate standard solution and the recoveries were calculated.
