# Supplementary material for: 2,4-D and IAA Amino Acid Conjugates Show Distinct Metabolism in Arabidopsis
Source: PLoS One. 2016 Jul 19;11(7):e0159269. doi: 10.1371/journal.pone.0159269 (PMC4951038; doi:10.1371/journal.pone.0159269)
Supplement: S3 Fig — (PDF) [file pone.0159269.s003.pdf]

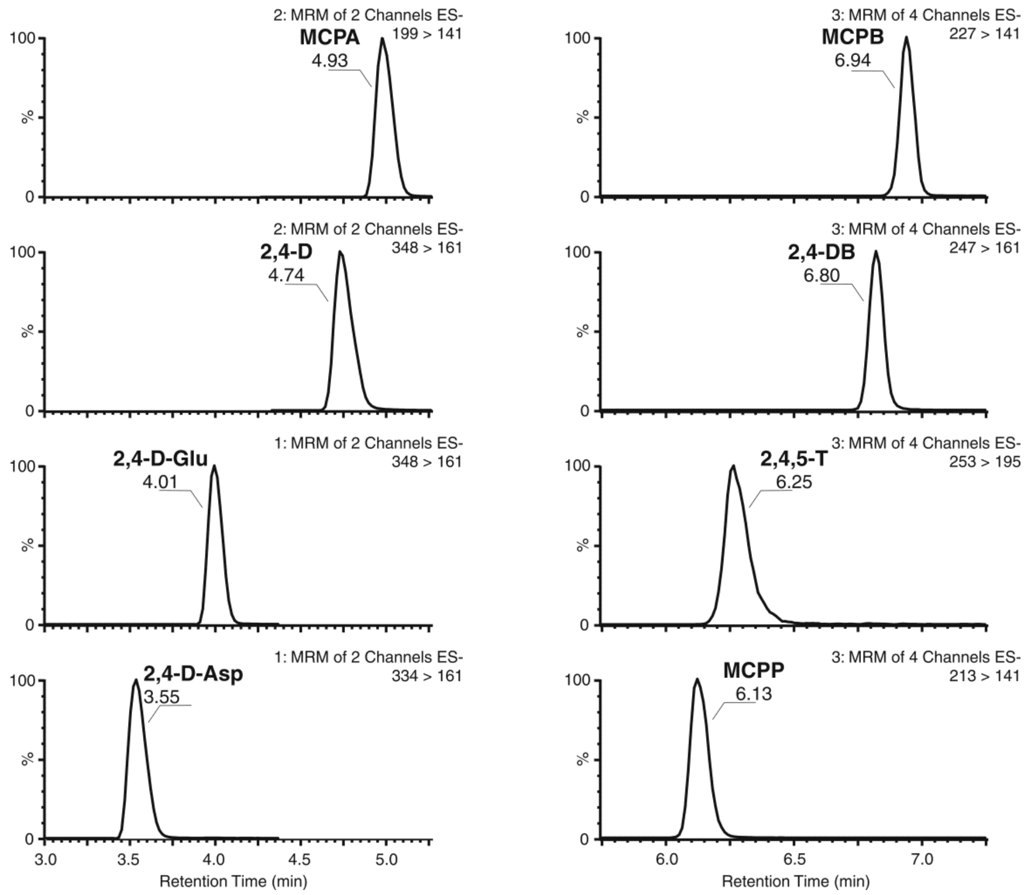

S3 Fig. Chromatographic separation of 2,4-D and its metabolites/structural analogues by UHPLC-(ESI–)-MS/MS. The figure shows the MRM chromatograms of 8 analytes represented by 10 pmol of each compound per injection using an Acquity UPLC® CSH™ C18 (2.1x100 mm, 1.7 µm) column.
