# Supplementary material for: 2,4-D and IAA Amino Acid Conjugates Show Distinct Metabolism in Arabidopsis
Source: PLoS One. 2016 Jul 19;11(7):e0159269. doi: 10.1371/journal.pone.0159269 (PMC4951038; doi:10.1371/journal.pone.0159269)
Supplement: S4 Fig — (PDF) [file pone.0159269.s004.pdf]

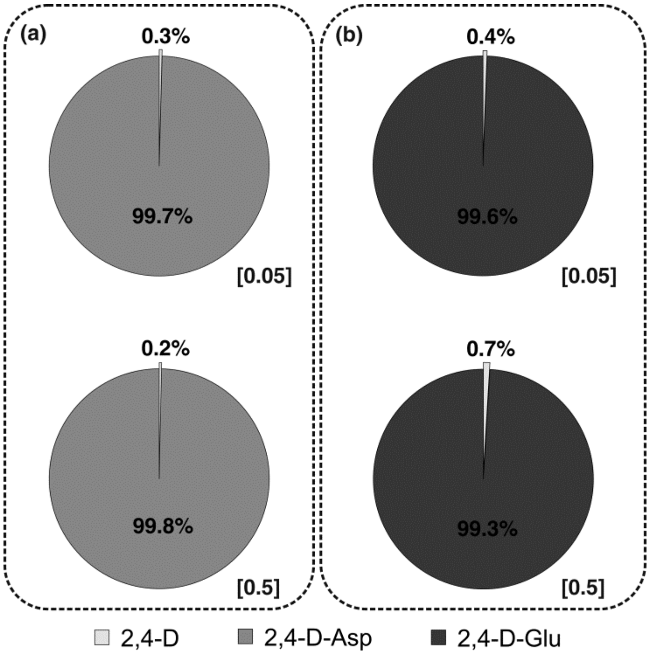

S4 Fig. One-week stability of 2,4-D-amino acid conjugates in growth media. Solid media (half-strength Murashige and Skoog, sucrose 1%, agar 0.7%, pH 5.7) supplemented with 2,4-D-Asp (a) and 2,4-D-Glu (b) at the indicated final concentrations (0.05 µM and 0.5 µM) were transferred to microcentrifuge tubes. After 7 days of incubation in the growth chamber (16/8 h of light/dark, 23°C), the media (200 µl) were melted in a microwave oven, diluted by a factor of 10, purified by the newly developed two-step purification procedure prior to the UHPLC-ESI(–)-MS/MS analysis and distribution (%) of free 2,4-D were calculated.
