# Supplementary material for: 2,4-D and IAA Amino Acid Conjugates Show Distinct Metabolism in Arabidopsis
Source: PLoS One. 2016 Jul 19;11(7):e0159269. doi: 10.1371/journal.pone.0159269 (PMC4951038; doi:10.1371/journal.pone.0159269)
Supplement: S5 Fig — (PDF) [file pone.0159269.s005.pdf]

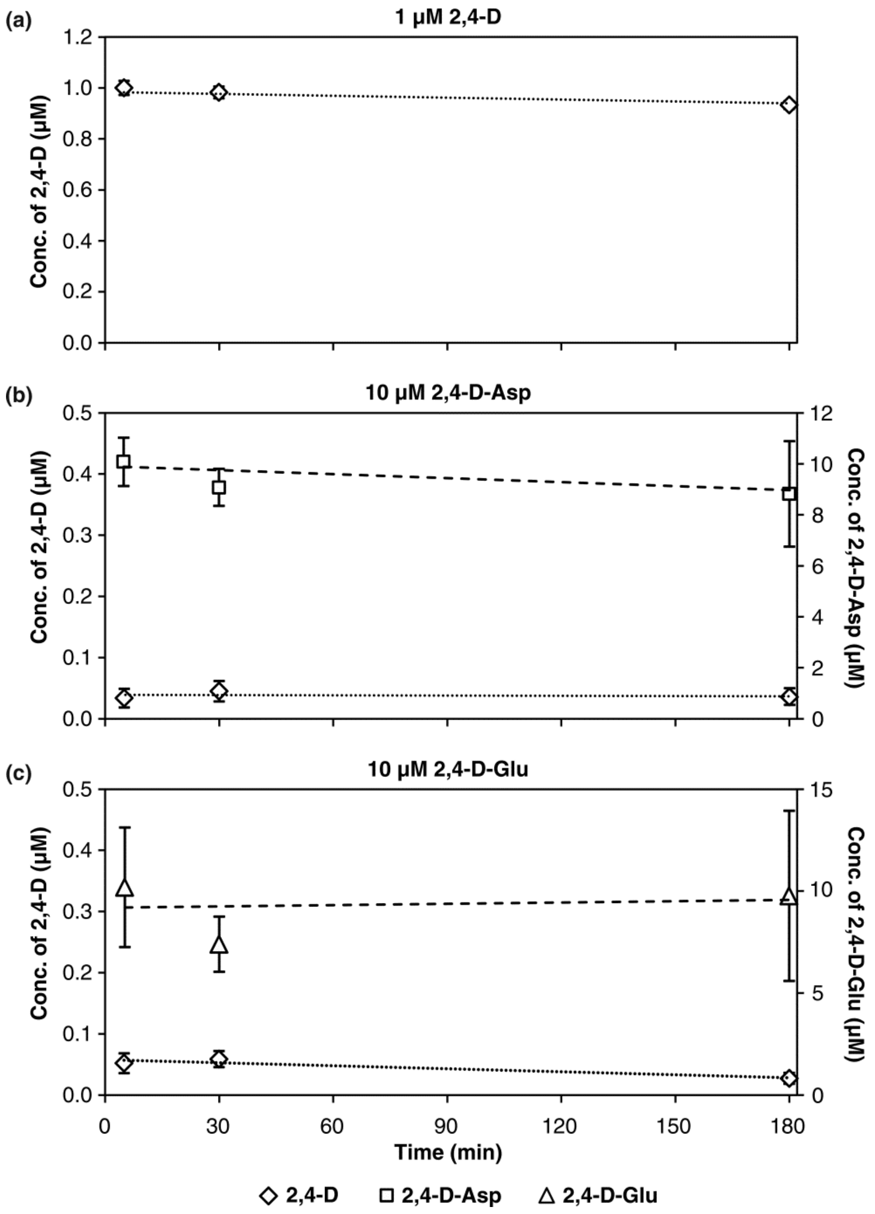

S5 Fig. Stability of 2,4-D, 2,4-D-Asp and 2,4-D-Glu in short-term chemical treatments. Solid media (half-strength Murashige and Skoog, sucrose 1%, agar 0.7%, pH 5.7) were treated with 2,4-D and 2,4-D-Asp/2,4-D-Glu at the final concentrations 1 µM and 10 µM, respectively, and transferred to microcentrifuge tubes. After 5 min, 30 min and 3 hours of incubation in the growth chamber (16/8 h of light/dark, 23°C), the media (200 µl) were melted in a microwave oven, diluted by a factor of 10 and analyzed by the UHPLC-ESI(–)-MS/MS. The concentration for all metabolites is in pmol g^{-1} FW. Samples were analyzed in four independent biological replicates, and error bars represent the SD. Statistical analysis was performed using ANOVA & comparison of means (Tukey's test) and showed no significant differences in means.
