# Supplementary material for: 2,4-D and IAA Amino Acid Conjugates Show Distinct Metabolism in Arabidopsis
Source: PLoS One. 2016 Jul 19;11(7):e0159269. doi: 10.1371/journal.pone.0159269 (PMC4951038; doi:10.1371/journal.pone.0159269)
Supplement: S1 Table — (PDF) [file pone.0159269.s006.pdf]

S1 Table. Preparation scheme and structural characteristics of 2,4-D-Asp. The purity of the synthesized compounds was confirmed by high performance liquid chromatography-liquid chromatography-diode array detection (Gold System, Beckman, Switzerland). The elemental composition of the prepared compounds was confirmed by HPLC-(ESI+)-HRMS (Q-TOF micro™ Waters MS Technologies, UK). Accurate masses were calculated and used for the determination of the elemental composition of the analytes with fidelity better than 3 ppm. NMR spectra were recorded on a JEOL ECA-500 spectrometer operating at frequencies of 500 MHz (^{1}H) and 125 MHz (^{13}C) and on a Bruker Avance 300 spectrometer with frequencies of 300 (^{1}H) and 75 MHz (^{13}C). Tetramethylsilane (TMS) was used as the internal standard.

Scheme 1

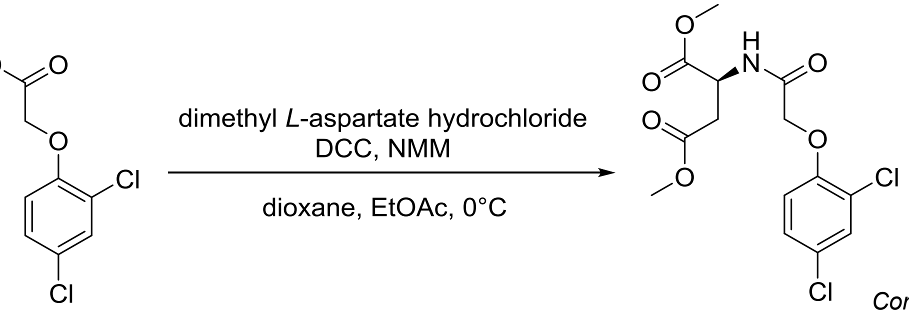

Compound 1: 2,4-D

Compound 2

Compound 2: **(*S*)-Dimethyl-2-(((2,4-dichlorophenoxy)acetyl)amino)butanedioate** (obtained in 94 % yield)

^{1}H-NMR (500 Hz, $CDCl_3$, δ): 7.75 (1H, d, *J* = 7.95 Hz, N-H), 7.40 (1H, d, *J* = 2.45 Hz, H-3'), 7.20 (1H, dd, *J* = 9.17 Hz, *J* = 2.45 Hz, H-5'), 6.82 (1H, d, *J* = 9.17 Hz, H-6'), 4.94 (1H, dt, *J* = 8.56 Hz, *J* = 4.28 Hz, H-2), 4.54-4.50 (2H, m, CH_2-O), 3.76 (3H, s, CH_3-O), 3.67 (3H, s, CH_3-O), 3.09 (1H, dd, *J* = 17.12 Hz, *J* = 4.28 Hz, H-1a), 2.87 (1H, dd, *J* = 17.12 Hz, *J* = 4.28, H-1b).

Scheme 2

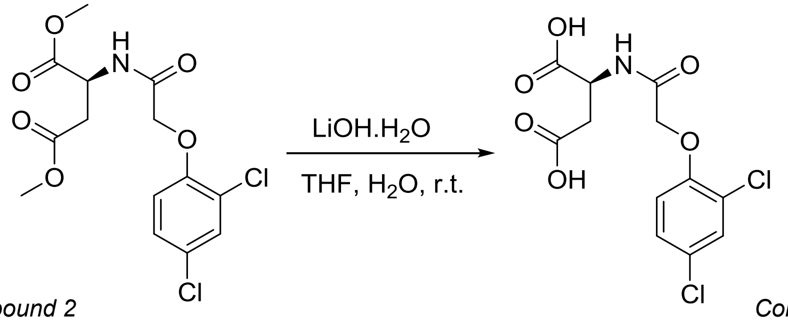

Compound 2

Compound 3

Compound 3: **(2-(2,4-dichlorophenoxy)acetyl)-*L*-aspartic acid** (obtained in 32 % yield)

^{1}H-NMR (500 Hz, DMSO-D_6, δ): 12.95 (2H, bs, CO_2H), 8.25 (1H, d, *J* = 7.95 Hz, N-H), 7.59 (1H, d, *J* = 2.45 Hz, H-3'), 7.33 (1H, dd, *J* = 9.17 Hz, *J* = 2.45 Hz, H-5'), 7.06 (1H, d, *J* = 9.17 Hz, H-6'), 4.66 (2H, s, CH_2-O), 4.58-4.54 (1H, m, H-2), 2.72-2.64 (2H, m, H-3).

^{13}C-NMR (125 Hz, DMSO-D_6, δ): 172.4 (CO_2H), 172.3 (CO_2H), 167.2 (C-NH), 152.7 (C-1'), 129.7 (CH-3'), 128.3 (CH-5'), 125.8 (C-4'), 123.0 (C-1'), 115,9 (C-6'), 68.0 (CH_2-O), 48.5 (CH-2), 36.3 (CH_2-1).

HRMS: (ESI–), $[M-H]^-$, *m/z* 333.9890 ($C_{12}H_{10}NO_6Cl_2$, Δ 1.5 ppm).

$^{24}\alpha_D$ = 15.3° (in H_2O + NaOH (2.2 equiv., c = 0.5 g/100ml)

Structural characteristics of $^{13}C_2$,^{15}N-2,4D-*L*-aspartic acid

^{1}H-NMR (500 Hz, DMSO-d_6, δ): 12.90 (2H, bs, CO_2H), 8.29 (1H, ddd, *J* = 92.6 Hz, *J* = 8.2 Hz, *J* = 3.6 Hz, N-H), 7.60 (1H, d, *J* = 2.4 Hz, H-3'), 7.33 (1H, dd, *J* = 8.9 Hz, *J* = 2.4 Hz, H-5'), 7.06 (1H, d, *J* = 8.9 Hz, H-6'), 4.66 (2H, dd, *J* = 147.6 Hz, *J* = 3.7Hz, CH_2-O), 4.62 (1H, bs, H-2), 2.71 (2H, bs, H-3).
