# Supplementary material for: 2,4-D and IAA Amino Acid Conjugates Show Distinct Metabolism in Arabidopsis
Source: PLoS One. 2016 Jul 19;11(7):e0159269. doi: 10.1371/journal.pone.0159269 (PMC4951038; doi:10.1371/journal.pone.0159269)
Supplement: S2 Table — (PDF) [file pone.0159269.s007.pdf]

S2 Table. Preparation scheme and structural characteristics of 2,4-D-Glu. The purity of the synthesized compounds was confirmed by high performance liquid chromatography-liquid chromatography-diode array detection (Gold System, Beckman, Switzerland). The elemental composition of the prepared compounds was confirmed by HPLC-(ESI+)-HRMS (Q-TOF micro™ Waters MS Technologies, UK). Accurate masses were calculated and used for the determination of the elemental composition of the analytes with fidelity better than 3 ppm. NMR spectra were recorded on a JEOL ECA-500 spectrometer operating at frequencies of 500 MHz (^{1}H) and 125 MHz (^{13}C) and on a Bruker Avance 300 spectrometer with frequencies of 300 (^{1}H) and 75 MHz (^{13}C). Tetramethylsilane (TMS) was used as the internal standard.

Scheme 3

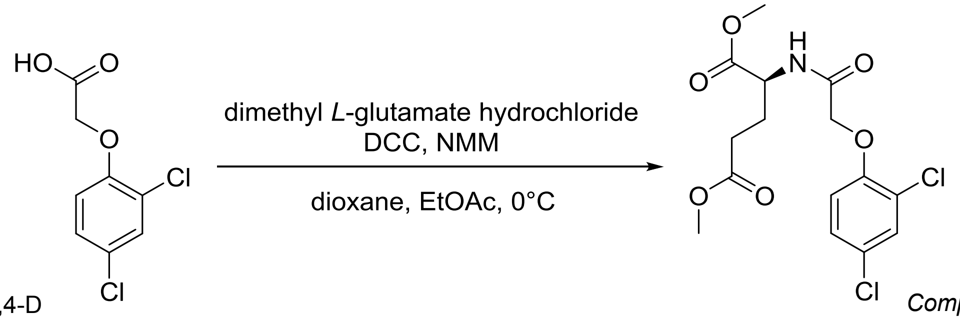

Compound 1: 2,4-D

Compound 4

Compound 4: **(*S*)-Dimethyl-2-(((2,4-dichlorophenoxy)acetyl)amino)pentanedioate** (obtained in 95 % yield)

H-NMR (500 Hz, $CDCl_3$, δ): 7.41 (1H, d, *J* = 2.45 Hz, H-3'), 7.39 (1H, bs, N-H), 7.21 (1H, dd, *J* = 8.56 Hz, *J* = 2.45 Hz, H-5'), 6.83 (1H, d, *J* = 8.5 Hz, H-6'), 4.72 (1H, dt, *J* = 7.95 Hz, *J* = 5.50 Hz, H-2), 4.56-4.48 (2H, m, CH_2-O), 3.76 (3H, s, CH_3-O), 3.65 (3H, s, CH_3-O), 2.43-2.25 (3H, m, H-4, H-3), 2.10-2.04 (1H, m, H-3).

Scheme 4

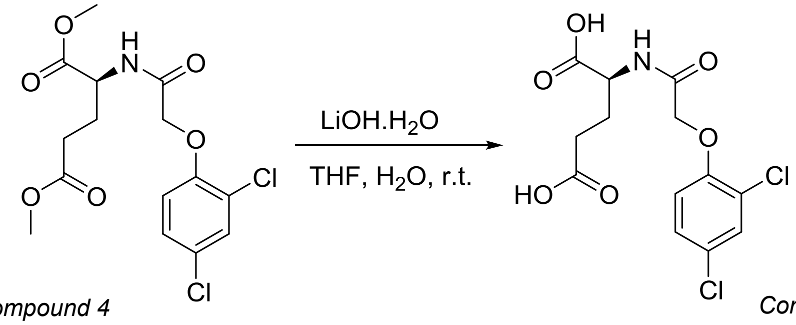

Compound 4

Compound 5

Compound 5: **(2-(2,4-dichlorophenoxy)acetyl)-*L*-glutamic acid** (obtained in 30 % yield)

^{1}H-NMR (500 Hz, DMSO-D_6, δ): 12.50 (2H, bs, CO_2H), 8.27 (1H, d, *J* = 7.34 Hz, N-H), 7.58 (1H, d, *J* = 3.06 Hz, H-3'), 7.32 (1H, dd, *J* = 8.56 Hz, *J* = 2.45 Hz, H-5'), 7.04 (1H, d, *J* = 9.17 Hz, H-6'), 4.63-4.67 (2H, m, CH_2-O), 4.27-4.23 (1H, m, H-2), 2.25 (2H, t, *J* = 7.34 Hz, H-4), 1.99-1.95 (1H, m, H-3), 1.84-1.80 (1H, m, H-3).

^{13}C-NMR (125 Hz, DMSO-D_6, δ): 174.3 (CO_2H), 173.3 (CO_2H), 167.4 (C-NH), 152.9 (C-1'), 129.8 (C-3'), 128.4 (C-5'), 125.5 (C-4'), 122.9 (C-2'), 115.8 (C-6'), 67.9 (CH_2-O), 51.6 (CH-2), 30.6 (CH_2-4), 26.9 (CH_2-3).

HRMS: (ESI–), $[M-H]^-$, *m/z* 348.0037 ($C_{13}H_{12}NO_6Cl_2$, Δ -1.4 ppm).

$^{24}\alpha_D$ = 12.8° (in H_2O + NaOH (2.2 equiv., c = 0.5 g/100ml)

Structural characteristics of $^{13}C_2$,^{15}N-2,4D-*L*-glutamic acid

^{1}H-NMR (500 Hz, DMSO-d_6, δ): 12.60 (2H, bs, CO_2H), 8.29 (1H, bd, *J* = 91.7 Hz, N-H), 7.59 (1H, s, H-3'), 7.33 (1H, d, *J* = 8.9 Hz, H-5'), 7.03 (1H, d, *J* = 8.9 Hz, H-6'), 4.68 (2H, dd, *J* = 147.9 Hz, *J* = 9.7Hz, CH_2-O), 4.28 (1H, bs, H-2), 2.26 (2H,t, *J* = 7.3 Hz, H-4), 2.02 (1H,bs, H-3α), 1.82 (1H,bs, H-3β).
