# Supplementary material for: 2,4-D and IAA Amino Acid Conjugates Show Distinct Metabolism in Arabidopsis
Source: PLoS One. 2016 Jul 19;11(7):e0159269. doi: 10.1371/journal.pone.0159269 (PMC4951038; doi:10.1371/journal.pone.0159269)
Supplement: S3 Table — (PDF) [file pone.0159269.s008.pdf]

S3 Table. Optimized UHPLC-(ESI–)-MS/MS parameters. The precursor and product ions of the studied compounds (MRM transition) and optimized cone/collision energies are listed for each analyte. The retention time stability, limits of detection, dynamic linear range, method linearity (correlation coefficients, R^2) are shown for UHPLC-ESI(–)-MS/MS analysis. The settings of the mass spectrometer in negative electrospray (ESI–) mode were as follows: Capillary Coltage, 3.0 kV; Cone Voltage, 21-30 V; Collision Energy, 10-22 eV; Low/High Resolutions, 3.0/15.0; Desolvation/Source Temperatures, 120/550°C. The gas flows were set to 0.21 ml/min of collision gas (Argon), 1000 l/h of desolvation gas and 70 l/h of cone gas (Nitrogen).

**Compound**	**MRM transition**	**Cone Voltage [V]**	**Collision Energy [eV]**	**Retention time [a] [min]**	**LOD[b] [fmol]**	**Linear range [pmol]**	R^2
**2,4–D**	219 > 161	21	10	4.742 ± 0.009	23	0.05–250	0.9995
**[$^2H_5$]-2,4-D**	224 > 164						
**2,4–D–Asp**	334 > 161	30	22	3.551 ± 0.011	20	0.05–250	0.9991
**[$^{13}C_2$,^{15}N]-2,4-D-Asp**	337 > 161						
**2,4–D–Glu**	348 > 161	25	22	4.014 ± 0.006	19	0.05–250	0.9989
**[$^{13}C_2$,^{15}N]-2,4-D-Glu**	351 > 161						
**2,4–DB**	247 > 161	17	9	6.801 ± 0.008	19	0.05–250	0.9986
**MCPA**	199 > 141	15	12	4.932 ± 0.004	14	0.05–250	0.9991
**MCPB**	227 > 141	18	9	6.941 ± 0.005	15	0.05–250	0.9975
**MCPP**	213 > 141	20	11	6.134 ± 0.008	10	0.05–250	0.9992
**2,4,5–T**	253 > 195	20	10	6.251 ± 0.006	150	0.5–250	0.9987

[a] Values are means ± SD (n = 10); [b] Lower Limit of Detection (LOD) defined as a signal-to-noise ratio of 3:1.
