# Supplementary material for: 2,4-D and IAA Amino Acid Conjugates Show Distinct Metabolism in Arabidopsis
Source: PLoS One. 2016 Jul 19;11(7):e0159269. doi: 10.1371/journal.pone.0159269 (PMC4951038; doi:10.1371/journal.pone.0159269)
Supplement: S4 Table — (PDF) [file pone.0159269.s009.pdf]

S4 Table. Characterization of monoclonal antibodies E2/G2 and immunoaffinity gel by 2,4-D structural analogues. Percentage of cross reactivity (CR) was calculated using the equation: CR(%) = IC_{50}(2,4-D)/IC_{50}(cross reactant)x100, where IC_{50} is the concentration of a competitor (cross reactant) resulting in 50% reduction of conjugate binding in direct ELISA system. For characterization of an immunoaffinity gel (IAG) with immobilised E2/G2 antibodies, mixtures of selected 2,4-D structural analogues with concentrations ranging from 1 to 300 pmol were applied onto 0.5 ml of IAG. Each spiking level was then determined by UHPLC-ESI(–)-MS/MS, compared with the concentration of appropriate standard solution and the recoveries were calculated. Values are means ± SD (n = 3); n.d. = not detected.

**Compound**	**CR (%)**	**Recovery (%)**					
		**1 pmol**	**5 pmol**	**10 pmol**	**50 pmol**	**100 pmol**	**300 pmol**
2,4–D	100.0	91.5 ± 7.9	75.3 ± 4.3	69.4 ± 4.9	59.2 ± 2.3	47.6 ± 7.9	32.6 ± 5.8
4-(2,4-dichlorphenoxy) butyric acid (2,4-DB)	0.2	n.d.	10.5 ± 3.2	14.7 ± 1.9	8.7 ± 0.7	2.5 ± 0.4	1.0 ± 0.3
2-methyl-4-chlorphenoxy acetic acid (MCPA)	8.04	32.4 ± 3.5	23.6 ± 1.0	15.3 ± 1.6	13.8 ± 1.5	4.0 ± 0.4	1.9 ± 0.3
4-(2-methyl-4-chlorphenoxy) butyric acid (MCPB)	0.9	n.d.	n.d.	n.d.	n.d.	n.d.	0.6 ± 0.1
2-(2-methyl-4-chlorphenoxy)propionic acid (MCPP)	<0.2	n.d.	n.d.	n.d.	n.d.	0.3 ± 0.1	0.2 ± 0.0
2,4,5-trichlorphenoxyacetic acid (2,4,5-T)	3.8	n.d.	n.d.	11.9 ± 2.8	9.0 ± 0.4	2.3 ± 0.5	1.1 ± 0.3
2,4-D-methylester (2,4-D-Me)	87.73						
2-chlorphenoxyacetic acid	0.9						
2-methyl-4-chlorphenoxyacetic acid	<0.2						
2-methyl-4,6-dichlorphenoxyacetic acid	<0.2						
2-methyl-6-chlorphenoxyacetic acid	<0.2						
4-chlorphenoxyacetic acid	0.9						
2,3-dichlorphenoxyacetic acid	1.6						
3,4-dichlorphenoxyacetic acid	2.7						
2-(2,4-dichlorphenoxy) propionic acid	0.4						
2,4-dichlorphenol	1.72						
2-methyl-4-chlorphenol	0.5						
2,4-dichloranisole	1.5						
3,4-dimethylphenol	<0.2						
pentachlorphenol	0.5						
