# Supplementary material for: 2,4-D and IAA Amino Acid Conjugates Show Distinct Metabolism in Arabidopsis
Source: PLoS One. 2016 Jul 19;11(7):e0159269. doi: 10.1371/journal.pone.0159269 (PMC4951038; doi:10.1371/journal.pone.0159269)
Supplement: S5 Table — (PDF) [file pone.0159269.s010.pdf]

S5 Table. Process efficiency and method validation (method precision and accuracy) for the ion-exchange and class-specific purification procedure. Extracts of 15 mg FW *Arabidopsis* leaves were spiked with three different concentrations of authentic standards (Low – 0.5 pmol; Medium – 5 pmol; High – 50 pmol) and known concentration of the appropriate internal labeled standards (10 pmol), purified by two-step isolation using the Oasis® MAX sorbent and the immunoaffinity gel, then directly analyzed by UHPLC-ESI(–)-MS/MS.

**Compound**	**Process Efficiency (%)** [a]	**Method precision (% RSD)** [b]			**Method accuracy (% bias)** [b]		
		**Low**	**Medium**	**High**	**Low**	**Medium**	**High**
**2,4–D**	66 ± 17	6.2	1.4	4.6	-3.6	3.3	14.6
**2,4–D–Asp**	16 ± 6	9.6	3.3	10.6	12.9	1.2	6.0
**2,4–D–Glu**	28 ± 8	8.2	2.3	7.3	18.3	4.6	-3.8

[a] Values are means ± SD (n = 12); [b] All analyses were performed in quadruplicate.
